# Technology readiness and UTAUT2 in e-wallet adoption in a developing country

**DOI:** 10.12688/f1000research.72853.1

**Published:** 2021-08-27

**Authors:** Min Yee Leong, Jing Hui Kwan, Lai Ming Ming

**Affiliations:** 1 Faculty of Management, Multimedia University, Cyberjaya, Selangor, 63100, Malaysia

**Keywords:** e-wallet, UTAUT2, cashless payment, technology readiness, Malaysia

## Abstract

**Background: **An e-wallet is a digital equivalent of a physical wallet which plays an essential role in payment system transformation. To embrace the concept of a cashless society, the Malaysian Government and central bank have taken various steps to encourage the adoption of e-wallets. Despite the seamless services offered by the e-wallet, it is yet to reach high-scale adoption in Malaysia. This study aims to investigate Malaysians’ readiness towards the e-wallet and their perceptions of it by employing the UTAUT2 model.

**Methods:** A total of 309 valid data were gathered and analysed with partial least squares structural equation modelling (PLS-SEM).

**Results:** The findings revealed that the respondents were confident about the new technology and tended to believe that e-wallet was somehow useful for them. The results also disclosed that e-wallet adoption intention was significantly influenced by performance expectancy, price value, facilitating conditions, and followed closely by social influence. Nonetheless, insecurity did not present significant impact on both performance expectancy and effort expectancy of e-wallet.

**Conclusions:** This study provides a substantial contribution to the knowledge domain by combining system-specific and individual-specific models in an e-wallet context. The outcomes of this study would also benefit e-wallet service providers and policymakers by delivering holistic insight into Malaysians’ readiness and adoption behaviour of the e-wallet.

## Introduction

The evolution of payment system is always in the spotlight and making the impossible come true such as cashless, card-less, and even contactless. Electronic wallets (e-wallets) appeared to be one of the famous examples of technology innovation in payment solutions. Back in 2011, the central bank, Bank Negara Malaysia proposed a ten-year masterplan called the Financial Sector Blueprint (FSBP) to provide a roadmap to further advance the country’s financial system ( Bank Negara Malaysia, 2011). One of the agenda items is to increase the offering and adoption of electronic payments (e-payments). As part of e-payments, the use of the e-wallet is welcomed in Malaysia as the country aims to move towards a cashless society. Bank Negara Malaysia has put various efforts in place such as guidelines on Interoperable Credit Transfer Framework (ICTF) and Electronic Money (E-Money) to encourage the adoption of e-wallets. In the year 2019, the government provided a one-off RM30 incentive for all eligible Malaysians in Budget 2020 and RM100 incentive for the youths in Budget 2021 to accelerate e-wallet adoption in the year 2020 ( Bank Negara Malaysia, 2019; Goverment of Malaysia, 2020).

Nevertheless, as reported by Nielsen Malaysia (2019), the e-wallet adoption rate in Malaysia is as low as 8%. It seems like Malaysians are not ready for a transformation despite the high financial literacy (95% of Malaysians are banked), high smartphone usage (91%) and Internet penetration (90.1%) in 2019 ( Department of Statistics Malaysia, 2019; Touch’N Go eWallet, 2019).

Given these facts, the Government and e-wallet service providers are facing challenges in attracting Malaysians to adopt e-wallets as a daily payment tool. Convincing people to use new technology is not a simple process as human thinking and behaviour are a complex process. Along this line, this study comprehensively integrated two models, namely technology readiness with Unified Theory of Acceptance and Use of Technology (UTAUT2), to understand Malaysian users’ readiness and perceptions towards the e-wallet.

### Technology readiness

Technology readiness is defined as a person’s propensity to embrace and use specific new technology for accomplishing life and work-related goals (Parasuraman, 2000, p. 308). It consists of four dimensions which can be categorized into two scopes, namely: positive enablers (optimism and innovativeness) and negative inhibitors (discomfort and insecurity) to measure human reactions ( Parasuraman, 2000a). Parasuraman (2000, p. 311) describes optimism as “a positive view of technology”; innovativeness as “A tendency to be a technology pioneer and thought leader”; discomfort as “a perceived lack of control over technology and a feeling of being overwhelmed by it”; lastly, insecurity as “distrust of technology and skepticism about its ability to work properly”.

Back in 2005, a new model named “TRAM” which is an integrated model of Technology Acceptance Model (TAM) and technology readiness was presented by Lin, Shih, Sher, & Wang (2005) to carry out a research regarding e-service adoption in Taiwan. The reason that led the authors to the combination is that TAM is system-specific whereas technology readiness is individual-specific, and therefore, they believed that they are interrelated. Extant research has incorporated technology readiness and technology adoption model to explain users’ readiness and perceptions toward financial technology ( Walczuch, Lemmink, & Streukens, 2007), electronic human resource management ( Erdoǧmu & Esen, 2011), electronic health record system ( Godoe & Johansen, 2012; Kuo, Liu, & Ma, 2013), web-based attendance management system ( Nugroho & Fajar, 2017) and mobile payment system ( Rahman, Taghizadeh, Ramayah, & Alam, 2017).

### Unified Theory of Acceptance and Use of Technology (UTAUT2)

UTAUT2 developed by Venkatesh, Thong, & Xu (2012), is the extension of UTAUT ( Venkatesh, Morris, Davis, & Davis, 2003). UTAUT2 consists of seven core constructs namely performance expectancy, effort expectancy, facilitating conditions, social influence, and three additional constructs: price value, habit, and hedonic motivation to explain one’s perceptions toward technology from the consumer view ( Venkatesh *et al*., 2012). Performance expectancy is defined as “the degree to which a person believes that using the system will help him or her to attain gains in job performance” ( Venkatesh *et al*., 2003, p. 447); effort expectancy is defined as “the degree of ease associated with the use of the system which reflects user perception of the difficulty when using a specific technology ( Venkatesh *et al*., 2003, p. 405); facilitating conditions is defined as “the degree to which a person believes that an organisational and technical infrastructure exists to support the use of the system” ( Venkatesh *et al*., 2003, p. 453); social influence is defined as “the degree to which a person perceives that important others believe he or she should use the new system” ( Venkatesh *et al*., 2003, p. 451); hedonic motivation is defined as “the pleasure or fun derived from technology use” ( Brown & Venkatesh, 2005; Venkatesh *et al.*, 2012, p. 161); lastly, price value is defined as “the trade-offs between the perceived benefits and cost of using a specific technology” ( Venkatesh *et al.*, 2012, p.161).

Compared to UTAUT, the improvements made in UTAUT2 have established a significant enhancement in describing the variability of behavioural intention (18% increase) and use behaviour (12% increase) ( Venkatesh *et al*., 2012). It can be said that UTAUT2 consists of the essences of the recognized theories. UTAUT3 is not considered in this study as the additional construct of UTAUT3: personal innovativeness is already involved in the technology readiness model.

### Research framework and hypothesis development

**Figure 1.**
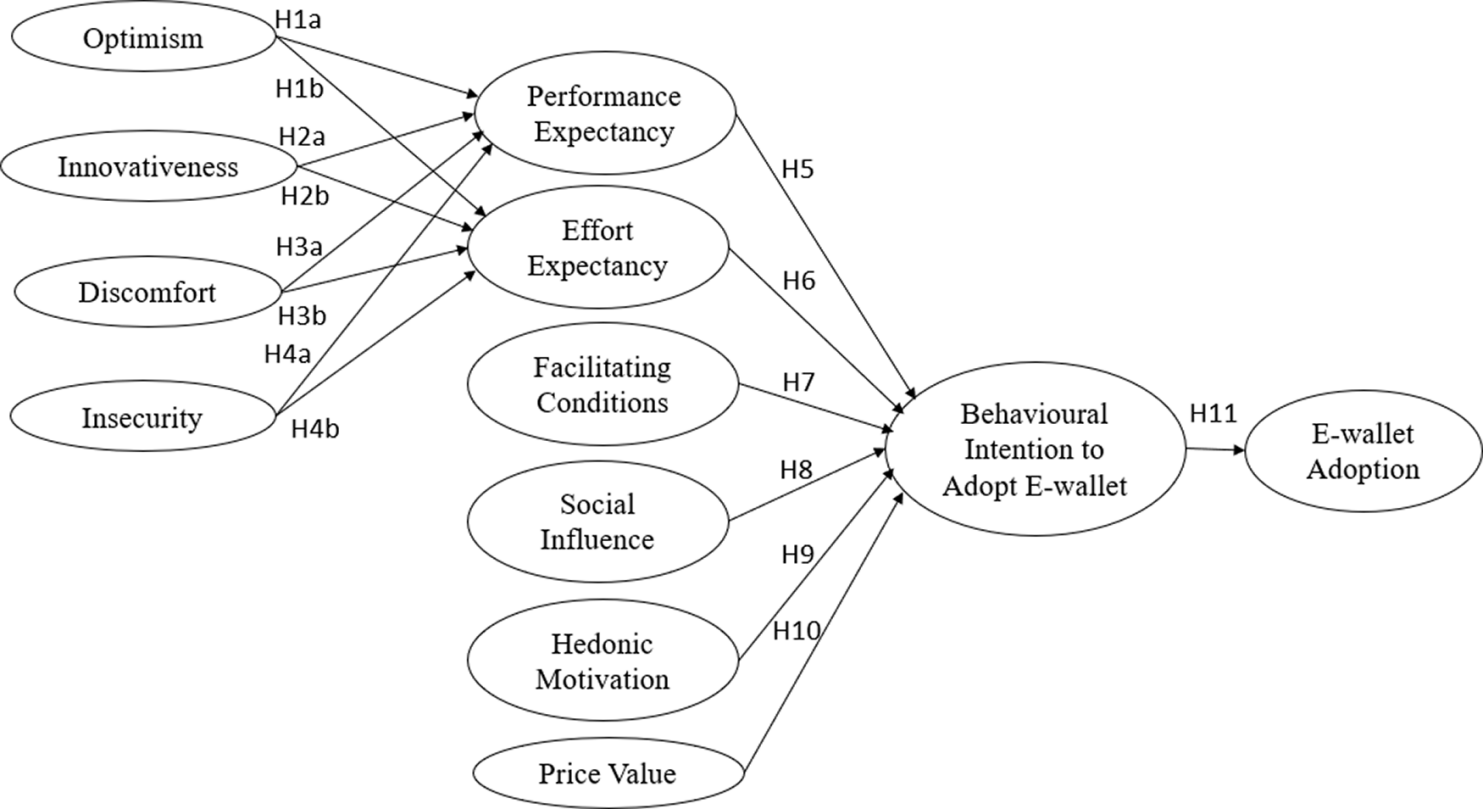
Research framework.

The proposed research framework is portrayed in Figure 1. The research framework proposed that the personality traits of technology readiness are interrelated with performance expectancy and effort expectancy from UTAUT2. Building on the literature of the TRAM model, this study postulated the following hypotheses:

H1: Optimism positively influences performance expectancy (a) and effort expectancy (b) of e-wallets among Malaysians.

H2: Innovativeness positively influences performance expectancy (a) and effort expectancy (b) of e-wallets among Malaysians.

H3: Discomfort negatively influences performance expectancy (a) and effort expectancy (b) of e-wallets among Malaysians.

H4: Insecurity negatively influences performance expectancy (a) and effort expectancy (b) of e-wallets among Malaysians.

Extant research has applied UTAUT2 to explain user adoption of e-wallets ( Tang, Lai, Law, Liew, & Phua, 2014), mobile payments ( Hussain, Mollik, Johns, & Rahman, 2019; Kalinic, Marinkovic, Molinillo, & Liébana-Cabanillas, 2019; Liébana-Cabanillas, Molinillo, & Ruiz-Montañez, 2019; Morosan & DeFranco, 2016; Oliveira *et al*., 2016; Sivathanu, 2019), mobile banking ( Alalwan *et al*., 2017), and mobile financial services ( Rahman *et al.*, 2017). The construct “habit” was eliminated as one should have rich experience in using such technology in order to allow examination of the “habit” role ( Alalwan, Dwivedi, & Rana, 2017; Oliveira, Thomas, Baptista, & Campos, 2016). Therefore, the following hypotheses were derived:

H5-H10: Performance expectancy (H5), effort expectancy (H6), facilitating conditions (H7), social influence (H8), hedonic motivation (H9) and price value (H10) positively influences behavioural intention to adopt e-wallets among Malaysians.

H11: Behavioral intention to adopt positively influences e-wallet adoption among Malaysians.

## Methods

The sample of this study is Malaysian e-wallet users. To acquire the empirical data needed to verify the research model, a structured questionnaire was adopted. The questionnaire consisted of three sections. Section A consisted of five screening questions related to the respondents’ e-wallet usage status to ensure that they were e-wallet users. The questions included have they ever used an e-wallet, what types of e-wallet are they using, when are they using e-wallets, how long have they been using e-wallets, and how frequently do they use e-wallets per month. Section B consisted of a 16-item Technology Readiness Index 2.0 scale adapted from Parasuraman & Colby (2014) to measure technology readiness and the last section consisted of a 26-item UTAUT2 scale adapted from Venkatesh *et al*. (2003, 2012). A seven-point Likert scale ranging from 1 (strongly disagree) to 7 (strongly agree) was considered.

Prior to the actual questionnaire distribution, a pilot study was carried out in the Multimedia Super Corridor (MSC) area to explore issues pertaining to the quality of the questions that could arise. A total of 49 valid responses were gathered and analysed using partial least squares structural equation modelling (PLS-SEM). The results reveal that one item from innovativeness construct need to be omitted because of low outer loadings. Hence, it was not considered in the actual questionnaire to ensure the consistency and accuracy of the items in measuring the constructs.

The actual data collection was conducted in Kuala Lumpur, Penang, Ipoh, Johor Bahru, and Melaka as they are the fastest-growing cities in the country, indicating high merchant acceptance of e-wallet payment which would allow the desired respondents to be precisely reached ( Gregory, 2018). The data collection involved conducting face-to-face communication with the respondents in 2019. To ensure the respondents were actual users of e-wallets, the targeted respondents for this study were practically reached by monitoring from afar their payment methods at the cashiers in the shopping malls whether e-wallet payments were used or not. Then, the researcher approached and invited these respondents to participate in this study. Written and verbal consent were sought from participants prior to conducting the survey. This survey had obtained approval number of EA1852021 from Research Ethics Committee of Multimedia University. The respondents were politely requested to answer the questions from a Google Form on a tablet. Within a two-month period (June and July 2019), 309 valid responses were gathered and used for further analysis. The data analysis was carried out using two software, namely IBM SPSS Statistics Version 22.0 and SmartPLS. IBM SPSS Statistics Version 22.0 was mainly employed for data screening and descriptive analysis of the profile of respondents usage frequency and percentage while SmartPLS was used for Partial Least Squares Structural Equation Modelling (PLS-SEM) evaluation.

## Results

### Respondents’ profile

IBM SPSS Statistics Version 22.0 was utilized to assess the demographic details such as gender, age, employment status and highest level of academic qualification of the sample respondents and their e-wallet usage status. It was found that the responses were made up of 120 male respondents and 189 female respondents. 74.4% of the respondents are from 18 – 34 years old range. With respect to employment status, 70.9% of them were employed full time. Among all the valid responses, 27.5% of them are degree holders and 21.7% of them qualified at “Sijil Pelajaran Malaysia or also known as SPM or below. It is worth noting that ‘Sijil Pelajaran Malaysia’ is equivalent to O-level certificate.

Based on the responses on the e-wallet usage status, Touch n’ Go e-wallet was found to be the most popular e-wallet with nearly 29.4% reporting using it. Majority of the respondents use e-wallet for transportation transaction (24.4%) and food and beverage transaction (21.4%). 35.9% of the respondents reported having one to six months usage experience. In terms of the frequency of e-wallet usage, 17.5% of the respondents were light users while 17.5% of the respondents were heavy users who used e-wallet more than eight times a month.

### Measurement model

To estimate and test the causal relations, partial least squares structural equation modelling (PLS-SEM) was considered because of its appropriateness in prediction purpose, exploratory study, and complex structural models (Hair, Ringle, & Sarstedt, 2011).

The reliability and validity of the constructs were first assessed. Based on the evaluation using SmartPLS, the outer loadings of all items above 0.708, except for INS1 (0.178), INS2 (0.388), and INS3 (0.553) of insecurity. The Average Variance Extracted (AVE) of insecurity was 0.329 which is under the acceptable value of 0.5. Hence, the indicator with the lowest loading, INS1 was deleted and the analysis was reconducted. The results in Table 1 showed that all the remaining indicators of insecurity achieved a satisfactory level for both outer loadings and AVE after removing INS1. The Composite Reliability (CR) values were also found to be above 0.867, indicating that the recommended CR value of 0.60 for exploratory research by Hair, Hult, Ringle, & Sarstedt (2016) was met.

The discriminant validity was analysed. It was found that all the Heterotrait-Monotrait ratios (HTMT) were found to be lower than 0.85, indicating the criteria of Kline (011) is fulfilled. Overall, the criteria of reliability and validity had been achieved at this stage of evaluation.

**Table 1.** Measurement model assessment.

Construct	Items	Loadings	CR	AVE
Optimism	OPT1	0.885	0.912	0.722
	OPT2	0.881		
	OPT3	0.811		
	OPT4	0.82		
Innovativeness	INNO1	0.865	0.919	0.79
	INNO2	0.895		
	INNO3	0.906		
Discomfort	DIS1	0.773	0.911	0.721
	DIS2	0.857		
	DIS3	0.903		
	DIS4	0.857		
Insecurity	INS2	0.759	0.867	0.687
	INS3	0.773		
	INS4	0.942		
Performance expectancy	PE1	0.883	0.938	0.791
	PE2	0.89		
	PE3	0.933		
	PE4	0.849		
Effort expectancy	EE1	0.925	0.961	0.859
	EE2	0.923		
	EE3	0.928		
	EE4	0.932		
Facilitating conditions	FC1	0.873	0.913	0.778
	FC2	0.902		
	FC3	0.872		
Social influence	SI1	0.953	0.973	0.923
	SI2	0.967		
	SI3	0.962		
Hedonic motivation	HM1	0.947	0.963	0.897
	HM2	0.957		
	HM3	0.936		
Price value	PV1	0.937	0.958	0.883
	PV2	0.942		
	PV3	0.94		
Behavioural intention to adopt	BI1	0.948	0.958	0.883
	BI2	0.926		
	BI3	0.946		

### Structural model

Before testing the hypotheses, the lateral collinearity issues were examined. All the Variance Inflation Factor (VIF) values were found to be below 2.503, suggesting that the issue of lateral collinearity is not a concern for this study.

In the path coefficients’ significance testing, bootstrapping was used to calculate the t-values with 5000 subsamples as the higher the number of subsamples, the higher the accuracy of the results ( Hair *et al*., 2016). As shown in Table 2, all the relationships were found to have t-values of at least 1.617, and thus, significant at 0.10 level of significance, except for H4a and H4b. In the coefficient of determination (R ^2^) assessment, it was found that all the R ^2^ values are above the threshold value of 0.26, indicating that the model is substantial.

**Table 2.** Structural model assessment.

Hypotheses	Standard beta	Standard error	t-value	Decision	R ^**2**^	f ^**2**^	Q ^**2**^
H1a	0.499	0.067	7.468 ***	Supported	0.332	0.321	0.238
H1b	0.310	0.075	4.121 ***	Supported	0.273	0.114	0.217
H2a	0.141	0.044	3.191 ***	Supported		0.025	
H2b	0.311	0.058	5.379 ***	Supported		0.114	
H3a	0.099	0.051	1.963 **	Supported		0.011	
H3b	0.169	0.071	2.377 ***	Supported		0.028	
H4a	0.073	0.066	1.105	Not supported		0.006	
H4b	−0.044	0.067	0.659	Not supported		0.002	
H5	0.290	0.067	4.299 ***	Supported	0.562	0.096	0.460
H6	0.109	0.068	1.617 *	Supported		0.011	
H7	0.146	0.055	2.642 ***	Supported		0.022	
H8	0.116	0.050	1.791 **	Supported		0.021	
H9	0.107	0.060	2.777 ***	Supported		0.011	
H10	0.176	0.063	9.59 ***	Supported		0.032	
H11	0.537	0.056	11.163 ***	Supported	0.288	0.405	0.209

Note: ***p < 0.01, **p < 0.05, *p < 0.10.

Next, the effect sizes (f ^2^) of the constructs are evaluated. The value of 0.02, 0.15, and 0.35 signify small, medium, and large effects ( Cohen, 1988). Based on the results, it was found that optimism had a medium impact on performance expectancy. The effect size values of performance expectancy, facilitating conditions, social influence, and price value indicated small effects on behavioural intention to adopt. The remaining constructs did not exhibit adequate effects on behavioural intention to adopt.

Predictive relevance was also assessed to predict the visible indicator of the constructs in the model which can be evaluated through Q ^2^ value ( Stone, 1974). The results showed that all the Q ^2^ values are higher than 0, implying that the model possesses a predictive relevance.

## Discussion

The results revealed that the respondents had strong positive feelings towards new technology, where they are hopeful about the usefulness of e-wallets. Additionally, the results showed that e-wallet adoption intention is strongly influenced by performance expectancy, price value, facilitating conditions followed closely by social influence, meaning that helpfulness, value gained, availability of facilitative assistance, and peer influence in using e-wallets are the decisive factors of usage intention among Malaysian e-wallet users. Nevertheless, the relationships between insecurity and performance expectancy and effort expectancy were found not to be significant. It can be said that even though some users feel anxious about new technology, it would not have any impact on the usefulness and ease of use of e-wallets.

The outcomes of this study offer valuable insights about the personality of Malaysian e-wallet users and their perceptions about e-wallets. Policymakers and e-wallet service providers could gain from the results of this study by learning and better understanding the way users behave and their needs and wants, and therefore, place necessary effort to enhance the use and adoption of e-wallets.

## Data availability

### Underlying data

Figshare: E_wallet_adoption.csv, https://doi.org/10.6084/m9.figshare.14871048.v1 ( Leong et al., 2021).

The project contains the following underlying data:

- E_wallet_adoption.csv

Data are available under the terms of the Creative Commons Zero “No rights reserved” data waiver (CC0 1.0 Public domain dedication).

## Author contributions

Kwan, J. H. and Lai, M. M. were involved in overall direction and planning and supervised the work. Leong, M. Y. developed the research framework, carried out the implementation and analysed the data. Leong, M. Y. wrote the manuscript with input from all authors.
