# Supplementary material for: Technology readiness and UTAUT2 in e-wallet adoption in a developing country
Source: F1000Res. 2021 Aug 27;10:863. [Version 1] doi: 10.12688/f1000research.72853.1 (PMC8453311; doi:10.12688/f1000research.72853.1)
Supplement: Peer review history

# Peer review: Reviewer response for version 1

**DOI:** 10.5256/f1000research.76460.r93282

**Published:** 2021-09-20

**Authors:** Peik Foong Yeap

**Affiliations:** 1 University of Newcastle, Callaghan, Australia; 2 Newcastle Australia Institute of Higher Education Pte Ltd, Singapore, Singapore

This article studies Malaysians’ readiness and perceptions towards the adoption of e-wallet using the Unified Theory of Acceptance and Use of Technology (UTAUT2) model. A sample of 309 valid data was collected and analysed with the partial least squares structural equation modelling (PLS-SEM). The results show that the adoption of e-wallet in this study is significantly influenced by performance expectancy, price value, facilitating conditions, and social influence. This study provides practical implications for e-wallet service providers and policymakers to enhance Malaysians’ readiness and adoption behaviour of e-wallet.

 The method of this study is appropriate and well designed. The data analysis and results have been explained clearly. The hypothesis has been tested and answered. The discussion has sound implications for practitioners and policymakers. 

 However, there are a few points that need to be further explained:

- The study adopted a structured questionnaire, but it was not mentioned clearly which questionnaire was adopted and the reasons why the questionnaire was adopted. It would be good to provide reasons and justification for the adoption of the structured questionnaire in the methods section and further attach it in the Appendix for reference.
- In addition, it would be good to justify the reason for using the seven-point Likert scale in this study.
- The findings in the discussion section could be further supported and discussed with findings from the previous studies in the literature.

Is the work clearly and accurately presented and does it cite the current literature?

Yes

If applicable, is the statistical analysis and its interpretation appropriate?

Yes

Are all the source data underlying the results available to ensure full reproducibility?

Yes

Is the study design appropriate and is the work technically sound?

Yes

Are the conclusions drawn adequately supported by the results?

Yes

Are sufficient details of methods and analysis provided to allow replication by others?

Yes

Reviewer Expertise:

Organizational behavior and sustainability, strategic management, inter-organizational collaboration, cross cultural inclusiveness, teaching and learning.

I confirm that I have read this submission and believe that I have an appropriate level of expertise to confirm that it is of an acceptable scientific standard.

# Peer review: Reviewer response for version 1

**DOI:** 10.5256/f1000research.76460.r93283

**Published:** 2021-09-03

**Authors:** Shaista Wasiuzzaman

**Affiliations:** 1 School of Business, Universiti Teknologi Brunei, Gadong, Brunei

The study investigates Malaysia's readiness and their perception towards the usage of an e-wallet as a payment tool. The paper is clear in that it follows the correct research process and the analysis conducted is suitable for the research objectives that have been formed for this research. Data is collected via a questionnaire distributed by the authors and the authors have provided the underlying data sources to ensure full reproducibility.

 However, the literature that the study refers to especially in the Introduction section is not up to date. The last research cited in this section is from the year 2017. It would be good if the researchers updated their literature review.

 Finally, the authors do not get into a detailed discussion of the results which may be due to the requirement of the journal. So to me, it seems lacking and the conclusion section is too short. I would have liked to see a discussion of the results with respect to the results of previous studies and based on the theory that was introduced earlier.

 I would have also liked to see the authors present the implications of the study to theory and practice. And also the limitations of their research with suggestions for future research directions.

Is the work clearly and accurately presented and does it cite the current literature?

Partly

If applicable, is the statistical analysis and its interpretation appropriate?

Yes

Are all the source data underlying the results available to ensure full reproducibility?

Yes

Is the study design appropriate and is the work technically sound?

Yes

Are the conclusions drawn adequately supported by the results?

Partly

Are sufficient details of methods and analysis provided to allow replication by others?

Yes

Reviewer Expertise:

Corporate finance, financial management

I confirm that I have read this submission and believe that I have an appropriate level of expertise to confirm that it is of an acceptable scientific standard.
